# Mobile Insertion Cassette Elements Found in Small Non-Transmissible Plasmids in *Proteeae* May Explain *qnrD* Mobilization

**DOI:** 10.1371/journal.pone.0087801

**Published:** 2014-02-04

**Authors:** Thomas Guillard, Antoine Grillon, Christophe de Champs, Céline Cartier, Janick Madoux, Béatrice Berçot, Anne-Laure Lebreil, Alain Lozniewski, Jacques Riahi, Véronique Vernet-Garnier, Emmanuelle Cambau

**Affiliations:** 1 EA 4687, UFR Médecine SFR CAP-Santé Université de Reims Champagne-Ardenne, Reims, France; 2 EA3964, PRES Sorbonne Paris Cité Université Paris Diderot-Paris 7, Paris, France; 3 Laboratoire de Bactériologie-Virologie-Hygiène, CHU Reims - Hôpital Robert Debré, Reims, France; 4 Laboratoire de Bactériologie, CHU Nancy - Hôpital Central, Nancy, France; 5 Laboratoire de Bactériologie, AP-HP Groupe Hospitalier Lariboisière - Saint Louis, Paris, France; University of Minnesota, United States of America

## Abstract

*qnrD* is a plasmid mediated quinolone resistance gene from unknown origin, recently described in *Enterobacteriaceae*. It encodes a pentapeptide repeat protein 36–60% different from the other Qnr (A, B, C, S and VC). Since most *qnrD*-positive strains were described as strains belonging to *Proteus* or *Providencia* genera, we hypothesized that *qnrD* originated in *Proteeae* before disseminating to other enterobacterial species. We screened 317 strains of *Proteeae* for *qnrD* and its genetic support by PCR. For all the seven *qnrD-*positive strains (4 *Proteus mirabilis,* 1 *Proteus vulgaris* and 2 *Providencia rettgeri*) the gene was carried onto a small non-transmissible plasmid, contrarily to other *qnr* genes that are usually carried onto large multi-resistant plasmids. Nucleotide sequences of the *qnrD*-bearing plasmids were 96% identical. Plasmids contained 3 ORFs apart from *qnrD* and belonged to an undescribed incompatibility group. Only one plasmid, in *P. vulgaris*, was slightly different with a 1,568-bp insertion between *qnrD* and its promoter, leading to absence of quinolone resistance. We sought for similar plasmids in 15 reference strains of *Proteeae*, but which were tested negative for *qnrD*, and found a 48% identical plasmid (pVERM) in *Providencia vermicola*. In order to explain how *qnrD* could have been inserted into such native plasmid, we sought for gene mobilization structures. *qnrD* was found to be located within a mobile insertion cassette (mic) element which sequences are similar to one mic also found in pVERM. Our conclusions are that (i) the small non-transmissible *qnrD*-plasmids described here may result from the recombination between an as-yet-unknown progenitor of *qnrD* and pVERM, (ii) these plasmids are maintained in *Proteeae* being a *qnrD* reservoir (iii) the mic element may explain *qnrD* mobilization from non-transmissible plasmids to mobilizable or conjugative plasmids from other *Enterobacteriaceae*, (iv) they can recombined with larger multiresistant plasmids conjugated in *Proteeae.*

## Introduction

Quinolones inhibit replication and transcription by inhibiting the bacterial type II topoisomerases, DNA gyrase and topoisomerase IV [1]. Quinolones attach to the DNA-topoisomerase complex, which becomes irreversible, leading to immobilization of the enzymes resulting in bacteriostasis and to the release of DNA double-strand breaks that activate the SOS system and produce the “poison” effect responsible for the intense bactericidal action of quinolones [2]. Fluoroquinolones (the main subgroup of quinolones) are currently among the most heavily prescribed antimicrobials in the world because of their pharmacodynamic and pharmacokinetic properties [1]. They are very potent, especially for treatment of urinary tract infections due to *Enterobacteriaceae* [1], [2], and as a consequence of their intense use, quinolone resistance rate has increased much for the last years [1], [3], [4]. Quinolone resistance mechanisms are multiple such as those reducing permeability of the bacterial wall, increasing efflux, reducing target affinity, producing inactivating enzymes and target protection proteins. Clinical resistance mostly results from the combination of several mechanisms [1]. Most of these mechanisms are chromosome-mediated [1], [2] but plasmid-mediated genes have been described for a decade [5]. *qnr* genes were the first plasmid-mediated quinolone resistance genes described in 1998 [6].

Qnr proteins are pentapeptide repeat proteins that protect DNA gyrase and topoisomerase IV from quinolone binding. Six families of plasmid-mediated *qnr* gene were described: *qnrA*, *qnrB*, *qnrC*, *qnrD* and *qnrS* [7]–[10]. Several alleles have been reported for each gene: 7 alleles of *qnrA*, 73 of *qnrB*, 1 of *qnrC*, 2 of *qnrD,* 9 of *qnrS* and 6 *qnrVC*. Alleles are numbered consecutively in a *qnr* library (http://lahey.org/qnrStudies, last update December 08, 2013). Most of *qnr* genes were detected in *Enterobacteriaceae*, where they located on large conjugative multi-resistance plasmids, such as pMG252 described in the original *Klebsiella pneumoniae qnrA1*-positive isolate [6]. The *qnrB* gene is the most prevalent in clinical bacterial strains, whereas *qnrS* gene is prevalent in environmental strains. Since *qnrA* alleles were found also as chromosome-borne in *Shewanella* spp. [11], this species was proposed to be a *qnrA* progenitor. Similarly, a *qnrB* allele was found in the chromosome of *Citrobacter* spp. [12], a *qnrS* and *qnrVC* allele in *Vibrionaceae* [10], [13]. *qnr* genetic environment usually showed mobile elements that can explain their spread from their progenitor, although they confer only a low level resistance to quinolones. Most of *qnrA* and *qnrB* genes were reported on large conjugative multi-drug resistant plasmids including aminoglycoside and beta-lactam resistance genes such as those encoding extended spectrum beta-lactamases, and in the vicinity of complex type 1 integrons [14], [15]. On the contrary, *qnrS* and *qnrD* genes were described on small non-conjugative plasmids, that may harbor *mob* genes allowing their mobilization [16], .

*qnrD* was first reported on a 4,270-bp (p2007057) plasmid in four *Salmonella enterica* isolates [7]. We subsequently described two *Providencia rettgeri* clinical isolates carrying *qnrD* on a 2,683-bp plasmid [18]. We hypothesized that *qnrD* can be originated from *Proteeae* or present in these bacteria as a reservoir. Our study determined how much *qnrD* is prevalent in recent clinical isolates of *Proteeae*, and investigated how *qnrD* could have disseminated to other bacterial species. We found seven nearly identical small *qnrD*-bearing plasmids, where *qnrD* is surrounded by sequences of mobile insertion cassette (mic) that may explain *qnrD* dissemination. Since the half part of the plasmid sequence excluding *qnrD*, was found in a native plasmid of *Providencia vermicola*, we think that *qnrD* might be present on the chromosome of a close, as yet unknown, *Proteeae* species and that its DNA recombination with *P. vermicola* native plasmid led to the small non-transmissible *qnr*-positive plasmid found in clinical isolates.

## Materials and Methods

### *qnrD* Detection in *Proteeae* Clinical Isolates

A total of 317 clinical isolates including 190 *Proteus mirabilis*, 59 *Morganella morganii*, 33 *Proteus vulgaris*, 20 *Providencia stuartii*, 14 *Providencia rettgeri* and 1 *Proteus penneri* were screened. They were isolated between 2008 and 2011 at five University hospitals (CHU Dijon, CHU Lariboisière Paris, CHU Nancy, CHU Pitié-Salpêtrière Paris, CHU Reims) from clinical specimens regardless antibiotic susceptibility. Fifteen reference strains of the tribe of *Proteeae* were purchased at the Collection de l′Institut Pasteur (CIP) (n = 13) and at the Leibniz-Institut DSMZ-German Collection of Microorganisms and Cells Cultures (Braunschweig, Germany) (n = 2) (Table 1).

**Table 1:** Reference strains of *Proteeae* harbored native plasmids but no *qnr* genes.

Organism	Strain collection designation	Plasmid content^a^
*Proteus mirabilis*	CIP 103181, ATCC 29906	No plasmid
*Proteus hauseri*	CIP 106868, ATCC 700826	No plasmid
*Proteus myxofaciens*	CIP 106872, ATCC 19692	No plasmid
*Proteus penneri*	CIP 103030, ATCC 33519	No plasmid
*Proteus vulgaris*	CIP 104989, ATCC 29905	No plasmid
*Providencia alcalifaciens*	CIP 82.90, ATCC 9886	14 kb (pPALC1, [35])
*Providencia rettgeri*	CIP 103182, ATCC 29944	5.5 kb (pPRET1, [35])
*Providencia rustigianii*	CIP 103032	150 Kb, 12 Kb
*Providencia stuartii*	CIP 104687, ATCC 29914	No plasmid
*Providencia vermicola*	CIP 108829, DSM 17385	3.6 kb (pVERM)
*Providencia heimbachae*	CIP 103031, ATCC 35613	No plasmid
*Providencia burhodogranariea*	DSM 19968, ATCC-BAA1590	−
*Providencia sneebia*	DSM 19967, ATCC BAA-1589	10.8 Kb (pPSN1), 7.6 Kb (pPSN2), 4.3 Kb (pPSN3) [35]
*Morganella morganii subsp. morganii*	CIP A231, ATCC 25830	No plasmid
*Morganella morganii subsp. sibonii*	CIP 103648, ATCC 49948	No plasmid

a Determined by agarose gel electrophoresis of plasmids extracted from the reference strains of *Proteeae* using the Kieser method. Control plasmids for size were pIP55 (130 kb), pIP135 (70.4 kb) and pGHS09-09 (2.6 kb).;

−, not done.

Detection of *qnr* genes was performed by multiplex real time PCR as previously described [19]. Briefly, after DNA extraction using the NucliSens easyMAG (bioMérieux, Marcy l′Etoile, France), multiplex real-time PCR was carried out using LightCycler® 480 (LC480) with HRM Supermix (Roche Molecular Diagnostics, Germany). The melt-curve analysis showed characteristic curves for each *qnr* gene family. All strains detected were confirmed to be *qnr*-positives by conventional PCR-sequencing.

The *qnrD*-positive isolates were compared for genome fingerprinting by Random Amplification of Polymorphic DNA (RAPD) using the Ready to Go RAPD analysis kit according to the manufacturer’s instructions (Amersham Biosciences).

### Characterization of *qnrD*-bearing Plasmids

Plasmid extraction was performed by the Kieser method from the *qnrD*-positive strains [20]. Plasmids harboring *qnrD* were sequenced on both strands using a Walk DS strategy [18]. Nucleotide sequences were aligned using the program BioEdit version 7.09.0 and BLAST searches using the National Center for Biotechnology Information website (http://www.ncbi.nlm.nih.gov) and the blastn algorithms**.** Putative promoters, ribosome binding site, and transcription start site were searched using BPROM (http://linux1.softberry.com) and Promoter Prediction by Neural Network [21].

Incompatibility groups of the plasmids were determined using the PCR-based replicon typing (PBRT) as described elsewhere [22]. Briefly, four multiplex PCR were used for the detection of A/C, T, FIIAs, W, N, FIB, L/M, I1-Iγ, X, HI2, FIA, and Y replicons. Replicons P, R, U, F, FIC, and K were detected by simplex PCR, as previously described, and replicons, FII1K, FII2K, NewXXX or also named ZK, LVPK and Amet (Table S1) as described by D. Decré and G. Arlet. The primer design was based on pGSH500 and pKNP4 (AJ009980 and CP000649 respectively), pLVPK (AY378100) and pK245 (DQ449578), and pMET-1 (EU383016) sequences.

### Search for *qnrD* and Small Non-transmissible Plasmids in Reference Strains of *Proteeae*

Plasmid extraction was performed by the Kieser method from the 15 reference strains and from 50 *qnrD*-negative strains randomly selected [20]. Based on the nucleotide sequences of the *qnrD*-plasmids characterized in the clinical isolates, six pairs of primers were designed corresponding to overlapping fragments (Table S1). Amplification products were visualized on 2% agar gel after electrophoresis and sequenced.

### Investigation of *qnrD* Transfer

Conjugation experiments were carried out using liquid and filter mating methods. Liquid mating experiments were performed in brain–heart infusion (BHI) broth with *E. coli* J53 azide-resistant [15], *P. mirabilis* ATCC 29906 Rif^R^ [23] and *E. coli* C600 Rif^R^ [23] as recipient strains. Donor and recipient cells in the logarithmic phase of growth were mixed (1 ml each) and incubated at 37°C for 3 hours without shaking. For filter conjugation, cultures (logarithmic phase of growth) of the donor and the different recipient cells were collected on a MF-type filter (Millipore, Molsheim, France). The hydrophobic edge membrane was incubated at 37°C on the surface of a BHI agar plate overnight. Transconjugants were selected on BHI agar plates containing sodium azide (100 µg/ml) or rifampin (250 µg/ml), with regard to the recipient strain, and nalidixic acid (50 µg/ml) or ciprofloxacin (0.015 µg/ml, 0.03 µg/ml, 0.06 µg/ml and 0.12 µg/ml) or ofloxacin (0.03 µg/ml, 0.06 µg/ml, 0.12 µg/ml, and 0.25 µg/ml).

The main capture systems (*intl1*, IS*CR*1, IS26, IS10, and ISE*cp*1) that have been associated with plasmid-mediated quinolone resistance determinants were detected using specific PCR (Table S1). *In silico* analysis was conducted for searching insertion sequence (IS) using IS Finder (http://www-is.biotoul.fr/is.html) and inverted repeats using REPFIND [24].

### Quinolone Resistance Testing

Since the *qnrD*-positive clinical isolates may have additional mechanisms conferring quinolone resistance, including chromosomal mutations, we determined the quinolone resistance of both the original *Proteeae* strains and the transformants obtained after transfer of *qnrD*-bearing plasmids into competent *E. coli* DH10B cells (Invitrogen, Cergy-Pontoise, France). Plasmid DNA was extracted from the *qnrD*-positive strains using the QIamp DNA mini Kit (Qiagen, Courtaboeuf, France) and introduced by electroporation (Gene Pulser, Biorad, Marnes la Coquette, France). Transformants were selected on BHI agar plates containing ciprofloxacin (0.015 µg/ml, 0.03 µg/ml, 0.06 µg/ml and 0.12 µg/ml) or ofloxacin (0.03 µg/ml, 0.06 µg/ml, 0.12 µg/ml, and 0.25 µg/ml). In order to distinguish transformants and quinolone resistant mutants growing on quinolone containing media, we sequenced the QRDRs in *gyrA* and *parC*, in addition to *qnrD* detection by PCR.

Minimal inhibitory concentrations (MICs) of nalidixic acid, ciprofloxacin, levofloxacin, moxifloxacin, norfloxacin, and ofloxacin were determined by the agar dilution method using Mueller–Hinton agar plates containing serially twofold-diluted antibiotics. Plates were inoculated with a Steers-type multiprong device with *ca*. 10^4^ CFU per spot, and were read after incubation for 18 hours at 37°C.

## Results

### Small Non-transmissible Plasmids are the Genetic Support of *qnrD* in *Proteeae* Isolates

*qnr* gene screening of the 317 clinical isolates resulted in eight (2.6%) *qnr*-positive strains with seven strains (87.5% of *qnr*-positive isolates) carrying *qnrD*: four *P. mirabilis* (DPROT11, DPROT104, DPROT189, and DPROT304), two *P. rettgeri* (DIJ09-518 and GHS09-09) and one *P. vulgaris* (DPROT78). The remaining *qnr*-positive isolate was a *P. mirabilis* isolate harboring *qnrA1* (DPROT289). The seven *qnrD* genes were 100% identical to the original gene described [7]. On the basis of epidemiological findings (Table 2) and RAPD profiles (data not shown), the isolates belonging to the same species (4 *P. mirabilis*, 2 *P. rettgeri*) were distinct strains.

**Table 2:** Epidemiological findings of the 7 *qnrD*-positive strains detected among 317 clinical isolates of *Proteeae*.

Strains	Hospital	Ward	Date of isolation	Specimen
*P. mirabilis* DPROT 11	CHU Reims	Nephrology	11/10/2010	Urine
*P. mirabilis* DPROT 104	CHU Reims	Diabetology	04/09/2010	Sputum
*P. mirabilis* DPROT 189	CHU Reims	Nephrology	02/18/2011	Urine
*P. mirabilis* DPROT 304	CHU Lariboisière-Paris	Long term care	02/02/2010	Urine
*P. vulgaris* DPROT 78	CHU Reims	Long-term care	12/12/2010	Urine
*P. rettgeri* DIJ09-518	CHU Dijon	Hematology	12/08/2008	Stool
*P. rettgeri* GHS09-09	CHU Pitié-Salpêtrière-Paris	Emergency room	10/09/2009	Urine

Conjugation assays, performed extensively, failed in transferring *qnrD* from clinical isolates to any of the recipient strains. From the seven *qnrD*-positive isolates, DNA extraction by the Kieser method showed several plasmids but a similar small plasmid of *ca.* 2.6 Kb in six isolates and one *ca.* 4 Kb in the remaining isolate. *qnrD*-plasmids were negative for known incompatibility groups.

Purified *qnrD*-plasmids were successfully transferred by electroporation into *E. coli* DH10B. Plasmid sequencing showed that six of the seven plasmids were of *ca.* 2.6-Kb long: pDIJ09–518a, pGHS09–09, pRS12–11, pRS12–104, pRS12–189, and pLRB12–304 being 2,683-bp, 2,683-bp, 2,863-bp, 2,683-bp, 2,656-bp and 2,658-bp long, respectively. They were carried, respectively, by two *P. rettgeri* clinical isolates previously described [18] and 4 clinical strains of *P. mirabilis*. The remaining plasmid (pRS12–78) was 4,236-bp long and was carried by a *P. vulgaris* isolate.

Quinolone MICs are presented in Table 3. Quinolone resistance conferred by the *qnrD*-bearing plasmids was similar for all plasmids, with a 60-fold increase in ciprofloxacin MIC. For the plasmid pRS12–78, transformation experiments on ofloxacin or ciprofloxacin, even at low concentration, selected S83L *gyrA* chromosomal mutants harboring pRS12–78. Consequently we cannot measure the exact level of resistance conferred by the plasmid. However, since the ciprofloxacin MIC was similar to that on a *gyrA* mutant [25], we concluded that the plasmid did not confer quinolone resistance, which is explained by the insertion between the putative promoter and the *qnrD* start codon (see below). No mutation in the QRDR of *gyrA* and *parC* were found in the other transformants.

**Table 3:** Resistance conferred by *qnrD-*bearing plasmids as measured by determination of minimal inhibitory concentrations (MIC) of quinolones on transformants with regard to parental strains of *Proteeae*.

Strains	MIC (µg/ml)					
	NAL	NOR	OFX	CIP	MXF	LVX
*P. mirabilis* DPROT11	>256	12	>32	>32	>32	16
*P. mirabilis* DPROT104	8	0.5	1	0.25	1	0.25
*P. mirabilis* DPROT189	>256	12	>32	>32	>32	16
*P. mirabilis* DPROT304	>256	8	>32	>32	>32	16
*P. rettgeri*GHS09-09	4	0.25	0.5	0.125	0.5	0.25
*P. rettgeri*DIJ09–518	>256	8	8	4	12	4
*P. vulgaris*DPROT78	>256	8	8	4	>32	4
*E. coli* DH10B	1	0.03	0.015	0.006	0.03	0.006
*E. coli* DH10B/pRS12-11	4	0.5	0.25	0.125	0.125	0.125
*E. coli* DH10B/pRS12–104	4	0.5	0.25	0.125	0.125	0.125
*E. coli* DH10B/pRS12–189	2	0.5	0.25	0.125	0.125	0.125
*E. coli* DH10B/pLRB12–304	4	0.5	0.25	0.125	0.125	0.125
*E. coli* DH10B/pGHS09-09	2	0.5	0.25	0.125	0.125	0.125
*E. coli* DH10B/pDIJ09–518a	2	0.5	0.25	0.125	0.125	0.125
*E. coli* DH10B/pRS78	16	0.125	0.125	0.03	0.06	0.006
*E. coli* DH10B/p2007057^a^	2	0.125	0.25	0.125	ND	ND

NAL, Nalidixic acid; NOR, Norfloxacin; CIP, Ciprofloxacin; OFX, Ofloxacin; LVX, Levofloxacin; MXF, Moxifloxacin; ND, no data.

a MICs reported by Cavaco *et al.* [7].

Nucleotide sequences (GenBank accession numbers: pDIJ09–518a, HQ834472; pGHS09-09, HQ834473; pRS12-11, KF364953; pRS12–104, KF364955; pRS12–189, KF364956; pLRB12–304, KF364957) of the six *ca.* 2.6-kb plasmids were 96% identical. These plasmids contain four putative open reading frames (ORFs) including *qnrD*, which we arbitrarily named ORF1 to ORF4. Two ORFs were similar to those found in p2007057 found in *S. enterica* [7] : the *qnrD* ORF1 (100% identity) and the ORF2 (97% identity). ORF2 encodes a hypothetical protein with 88% similarity to a hypothetical protein previously described (accession number AF448250). ORF3 and ORF4 are also hypothetical proteins with no significant matches.

**Figure 1:**
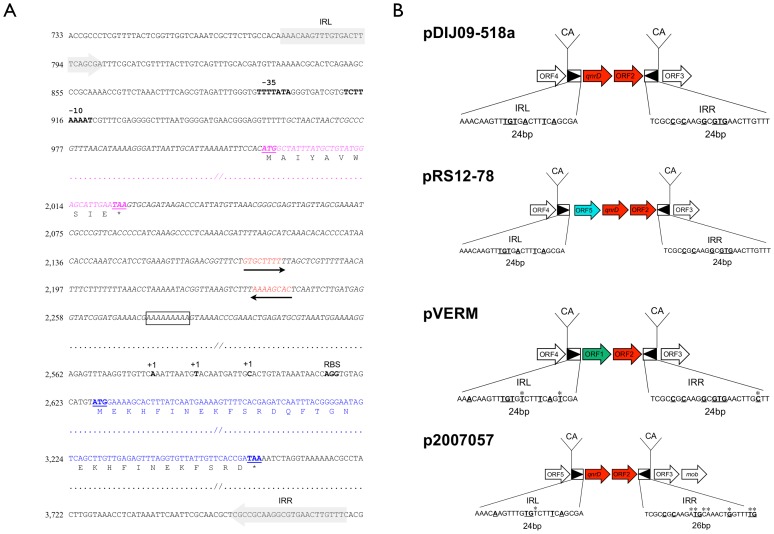
*qnrD* is located within a mobile insertion cassette (mic) element. A. Sequence analysis of the mic element including *qnrD* from the sequence of the plasmid pRS12–78, which contains a 1,603 bp insert (ORF5) upstream from *qnrD*. The nucleotide numbering is shown on the left-hand side. Putative -35, -10 promoter regions as well as the ribosome binding site (RBS) and the putative transcription start sites (+1) of *qnrD* are in bold. The start and the stop codons of *qnrD* (in blue) and of the ORF5 (in magenta) are underlined. For the termination site of ORF5, two black arrows indicate inverted repeats, and the string of “A’s” is boxed. The inverted repeats (IRL and IRR) bracketing the mic element are boxed as arrows shaded in grey. In pRS12–78, *qnrD* is far from its own promoter due to the insertion of ORF5. B. Comparison of the mic elements found in the plasmid, pDIJ09–518, pRS12–78, pVERM and in p2007057. Open reading frames (ORFs) are indicated by horizontal arrows. White arrows are identical ORFs harbored by the different plasmids beyond the mic. Red arrows are identical ORFs harbored by the different plasmids within the mic. The right and left inverted repeats (IRR and IRL) are indicated as black triangles with the duplication sites (CA). Nucleotide sequences of the IRL and IRR are outlined and the nucleotides which differed between them are in bold type and underlined. Asterisks show nucleotide differences of IRs with regard to those in pDIJ09–518a.

Plasmid pRS12–78 (GenBank accession number KF364954) was identical to the 2.6-Kb plasmids but with an additional 1,568 bp insert between *qnrD* and its promoter (Figure 1A). BLAST comparison highlighted a putative ORF5 exhibiting 76% identity with a hypothetical protein (GenBank accession number ADR29581) close to the ATPase domain of the *E. coli* ParA protein. Moreover, at 147 nucleotides downstream the stop codon of ORF5, the presence of inverted repeats followed by a short string of A’s suggested a factor-independent termination site.

Interestingly, among the randomly selected *qnrD*-negative strains for plasmid characterization using Kieser extraction, none of them harbored small plasmids (data not shown).

### *qnrD* is Included in a Mobile Insertion Cassette

*In silico* analysis did not show any gene involved in mobilization and specific PCRs for *intl1*, IS*CR*1, IS26 and ISE*cp*1 were negative. We also looked for mobile insertion cassette (mic) elements that are similar to transposons. A mic element is bracketed by two inverted repeats without any transposase-encoding gene found nearby and duplication of the target site is usually observed indicating the transposition. In the 2,683-bp long plasmids, *qnrD* was localized within a 1,519-bp “mic” element, bracketed by two 24-bp imperfect inverted repeats (IR) with six mismatches (Figure 1B). No transposase-encoding gene was found. Acquisition of the mic-*qnrD* element was likely the result of a transposition process because it is bracketed by a 2-bp duplication of the target site (CA). The mic-*qnrD* element described here carries the necessary elements for expression of the *qnrD* gene: (i) promoter sequences including a –35 box (TTTTATA) and a –10 box (TCTTAAAAT) separated by 11-bps, (ii) and a Shine-Dalgarno sequence (AAAAGGG) upstream on the *qnrD* initiation codon (ATG).

In the 4,686-bp long plasmid pRS12–78, *qnrD* is a part of a 3,072-bp “mic” element given that the ORF5 described above was inserted between *qnrD* and a putative promoter (Figure 1A). Although this second mic-*qnrD* element is slightly different in length, it has the same characteristics.

### Reference Strains of *Proteeae* Carried Native Plasmids but no *qnrD* Gene

*qnrD* was not detected by multiplex or simplex real-time PCR in the fifteen reference strains of *Proteeae* but plasmids were found in most of the reference strains (Table 1). These plasmids did not belong to any known incompatibility group detected using the PBRT method [22].

Overlapping PCRs designed on the sequence of pDIJ09–518a and performed using template DNA of the *Proteeae* reference strains showed that one small plasmid of *P. vermicola*, pVERM, carried a fragment exhibiting 89% identity with the sequence of pDIJ09–518a. pVERM was linearized using *AclI* restriction enzyme. After gel extraction, the sequence was determined using a walk DS strategy on the amplification product obtained with primers pDIJ-2558F and orf2–3′R. pVERM is a 3,682-bp long plasmid containing a 1,794-bp region sharing more than 98% identity with the *qnrD*-plasmids. In this region, we identified three ORFs similar to ORF2, ORF3 and ORF4 described in pDIJ09–518a. BLAST of the 1,888-bp remaining region showed a 267-bp ORF1 (named pVERM_ORF1 in the following) covering 76% of a putative protein described in *Vibrio brasiliensis* (accession number ZP_080099857.1) with 31% similarities. Two 24-bp long imperfect IR, with eight mismatches, and CA duplications were found to bracket pVERM_ORF1 and pVERM_ORF2 leading to identify another mic element. The 2,664-bp long mic element harbored by pVERM is different from mic-*qnrD* for 1,145 bps containing pVERM_ORF1 and lacking *qnrD* (Figure 1B). Nonetheless, IRR and IRL are similar to those bracketing mic-*qnrD* on the *qnrD* plasmids such as pDIJ09–518a with 2 and 1 single nucleotide differences for IRLs and IRRs respectively (Figure 1B). No known mobilization structure such as IS were identified in this 2,664-bp long mic element.

## Discussion

We investigated the emergence of the new plasmid-borne quinolone resistance gene, *qnrD* in *Proteeae* clinical bacterial isolates. The *qnr* genes are encoding a group of pentapeptide repeat proteins recently discovered but certainly present on bacterial chromosomes long before quinolones were used clinically. They emerged 10 years ago, following the 1990–2010 worldwide increase in fluoroquinolone usage [3]. *qnrD* was initially described in *Salmonella enterica* Kentucky and Bovismorbificans isolates [7], our investigation showed that *qnrD* was the *qnr* gene mainly present in isolates belonging to the *Proteeae* tribe (*Proteus*, *Providencia* and *Morganella* genera) since the screening of all *qnr* genes in 317 Proteeae clinical isolates resulted in 8 *qnr*-positive strains but seven (87.5%) positive for *qnrD.* This unexpected result converges with data in GenBank where among 38 sequences of *qnrD* half were reported in *Proteeae* (Table S2, last update October 30, 2013). We consequently hypothesized that *Proteeae* are the reservoir of *qnrD* genes.

In *Proteeae* clinical isolates, we found *qnrD* on small *ca.* 2.6-kb non-transmissible plasmids, in contrast with what was mainly described for other *qnr* genes carried by large conjugative plasmids [5]. Although *qnrD* was also reported to on such large conjugative plasmids, none of these plasmids was well characterized [26]–[29]. All the 15 *qnrD*-bearing plasmids found in *Proteeae* (the 7 from our study and 8 plasmids from GenBank sequences, Table S2) have the same size (2,683-bp) and have nearly identical sequences, which is in favor of a common origin or ancestor [30]–[32]. The only exception found during our study is the plasmid pRS12–78 that presented a plasmid scaffold identical to the other characterized plasmids but with a 1,568-bp insertion corresponding to a putative additional ORF. By contrast, the plasmids bearing *qnrD* found in strains of *Enterobacteriaceae* other than *Proteeae* are 4,270 bp long with sequences nearly identical to p2007057 described in *S. enterica*. These latter plasmids usually harbor a *mob* gene that may explain their transfer. The two types of plasmids carrying *qnrD* show a highly conserved backbone, but the smallest are not transmissible. This was previously described for *qnrS*, as it was described on a 10,066-bp non-conjugative but mobilizable plasmid in *S. enterica* Typhimurium [16]. It was suggested that this plasmid results from the recombination of a *qnrS1* conjugative plasmid from *S. enterica* Infantis (pINF5) and a small mobilizable plasmid from *E. coli* (pEC278). The *qnrD*-2,683-bp plasmids were stable in *E. coli* DH10B after electroporation, indicating a host range including other species of *Enterobacteriaceae*. However, the gene encoding the replicase is probably different to what is usually found since the incompatibility group cannot be assessed. Non classic replication systems were recently reported for some small plasmids in *Proteeae* [18], [33] and we share the view of Galac *et al.* that cryptic plasmids in *Proteeae*, including the small *qnrD*-plasmids, may use a not yet characterized replication method [33]. Since reference type strains of *Proteeae* did not harbor *qnrD*, we sought for similar plasmid backbone in their constitutive DNA. We found, in a reference strain of *P. vermicola,* a native plasmid that shares 1,794 bps with more than 98% identity with our *qnrD*–bearing plasmids.

We described here *qnrD* as located in a mobile insertion cassette (mic) element. Mic elements were first described in *Bacillus cereus* (MIC*231*A1) as a DNA cassette able to be mobilized in *trans* by the IS*231*A transposase [34]. MIC*231*A1 contains an active D-stereospecific endopeptidase (*adp*) gene instead of a transposase. Further studies conducted on *B. cereus* showed the presence of antibiotic resistance determinant such as a fosfomycin resistance gene in MIC*231*D, described as a novel active composite cassette [35]. It was then proposed mic element could be also a vehicle for antibiotic resistance genes beside class 1 integrons where multiple antibiotic cassettes can be inserted. With regard to plasmid mediated quinolone resistance genes, *qnrS2* was described in a mic element in a strain of *Aeromonas punctata* isolated in water sampled from the River Seine in Paris [36]. Unfortunately, as reported by Cattoir *et al*., no transposases were found, on the basis of the IR nucleotide sequences, suitable for demonstrating trans-transposition in an *in vitro* assay [36]. *qnrA* genes were mainly associated with IS*CR1* at the vicinity of complex class 1 integrons which include various other resistance determinants (*e.g. bla* genes or *aac(6′)-Ib-cr*). *qnrB* genes were also described beside class 1 integrons, but associated with IS*Ecp1* and ORF1005, another type of inserted sequences [37], [38]. Unlike *qnrA* and *qnrB*, *qnrS* genes were mainly associated with structure such as IS*Ecl2*, IS26 and mic structure [16], [36], [39], and were not reported as part of complex integrons. These gene-capture systems explain the mobilization of *qnr* genes from their progenitors to self-transmissible plasmids leading to their widespread in *Enterobacteriaceae*.

**Figure 2:**
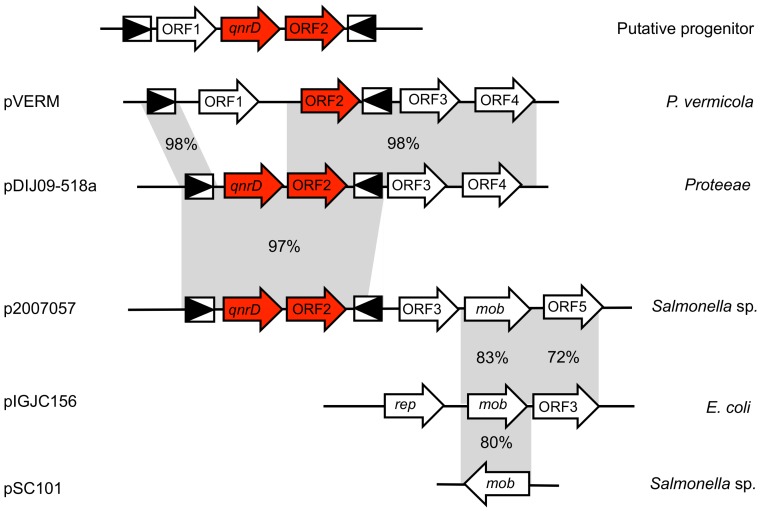
*qnrD* may have emerged and disseminated from *Proteeae* to *Enterobacteriaceae* using mic elements and a recombination event with the native PVERM plasmid. Comparison of the plasmid structures of pDIJ09-518a from *P. rettgeri*, pVERM from *P. vermicola*, p2007057 from *S. enterica* Bovismorbificans, pIGC156 from *E*. *coli* and pSC101 from *S. enterica* Typhimurium. The open reading frames are shown as arrows with the arrowhead indicating the direction of transcription. Red color is used to shade arrows in the *qnrD*-mic element. Grey shading shows the areas of homology between the plasmids.

We think that *qnrD* was imported from a *Proteeae* species and inserted in pVERM through mic mobilization (Figure 2). Further recombination, insertion or deletion events may have occurred after the mic-*qnrD* mobilization including *qnrD* deletion, resulting in the plasmids pVERM and pDIJ09-518a reported here. Indeed, the IRs found in the mic-*qnrD* plasmids from *Proteeae* clinical strains are more similar to those found in pVERM than those found in plasmids in *S. enterica*. pVERM differed from *qnrD*-plasmids by 1,888 bps located inside the very same lacking *qnrD* mic. This *qnrD*-lacking-mic element found in pVERM is 1,145 bp longer than mic-*qnrD* and carried a pVERM_ORF1 present neither on pDIJ09-518 nor on p2007057. While mic-*qnrD* IRR and IRL showed seven unmatched nucleotides for pVERM *vs* six for mic-*qnrD*, more importantly a comparison of IRLs between mic-*qnrD* and pVERM found only 2 SNPs and only 1 SNPs was found comparing both IRRs. Finally, IRs in both *qnrD*-plasmid and pVERM were 24-bp long. (Figure 1A). Moreover, none of the 50 *qnr*-negative clinical strains, randomly selected, harbored such small plasmids. Therefore, our current hypothesis is that pVERM could be the ancestor plasmid where *qnrD* was later deleted. Alternatively, pVERM could be a *qnrD*-plasmid resulting from another mic-*qnrD* harbored by an unknown *qnrD* progenitor, from which *qnrD* was subsequently lost.

The *qnrD* progenitor is not yet identified. For the other *qnr* genes, progenitors are mainly bacteria living in the environment (*Shewanella* spp., *Citrobacter* spp., and *Vibrio* spp) where *Proteeae* are also present [13], [36], [40], [41]. The *Proteeae* could have acquired *qnrD* from its progenitor by recombination between an as-yet-unknown bacterial source of *qnrD* and the cryptic plasmid pVERM harbored by *P. vermicola* leading to small non-mobilizable 2,683-bp long plasmids carrying *qnrD* embedded in a mic-*qnrD* element (Figure 2). Given that all plasmids of about 4,270 bps such as p2007057 (e.g. *P. mirabilis* isolates CGP180 and CGH40 described by Zhang *et al.*) also harbored a mic-*qnrD* with a conserved 24-bp IRL and a modified 26-bp IRR showing 1 and 7 nucleotide differences, respectively, with regard to 2,683-bp *qnrD*-plasmids, we think that the mic*-qnrD* element could have been later inserted by *trans* transposition in a mobilizable plasmid such as the 4,270-bp *qnrD*-plasmid p2007057. It is also possible that the mic-*qnrD* was transferred from a common progenitor to both *Proteeae* and *Salmonella* spp. in parallel onto two different types of plasmids in which the IRs sequences evolved and diverged especially for the IRR.

Finally, we investigate the quinolone resistance conferred by the *qnrD*-bearing plasmids. The MICs of fluoroquinolones were similar and comparable to MICs previously reported for p2007057, except for the plasmid pRS12–78. In this plasmid, the insertion of a 1,568-bp long sequence, can hamper *qnrD* expression from a putative promoter then located far from *qnrD*. Although the level of resistance is rather low for classical quinolones such as nalidixic acid, it can reach 60-fold increase in the MIC of ciprofloxacin. Such an increase can cause failure in experimental model of pyelonephritis and in pulmonary infections [42]–[44]. Further studies are needed to investigate the fitness cost of *qnrD* harbored by these small plasmids, but as described for *qnrA3* [45], *qnrD* could contribute to a fitness gain leading to the emergence of *qnrD*-bearing plasmids even in the absence of quinolones.

## Supporting Information

Table S1 List of primers used in this study.
(DOC)

Table S2 List of *qnrD* deposited in GenBank.
(DOC)
