# Supplementary material for: Mobile Insertion Cassette Elements Found in Small Non-Transmissible Plasmids in Proteeae May Explain qnrD Mobilization
Source: PLoS One. 2014 Feb 4;9(2):e87801. doi: 10.1371/journal.pone.0087801 (PMC3913671; doi:10.1371/journal.pone.0087801)
Supplement: Table S1 — List of primers used in this study. (DOC) [file pone.0087801.s001.doc]

**Table S1.** List of primers used in this study.

| Primer | Primer sequence (5’→3’) | Position | Size of amplified product (bp) | Ref |
| --- | --- | --- | --- | --- |
| pDIJ-334F ^a^ | GCAGAATAACGGGTGAAATAAT | 334-355 | 644 | This study |
| pDIJ-977R ^a^ | CGGGCGAGTTAGTTAGC | 977-961 |  | This study |
| pDIJ-873F ^a^ | CTTTCAGCGTAGATTTGGG | 873-891 | 614 | This study |
| pDIJ-1486R ^a^ | GTTCGCACTTTTCTAATATGACT | 1486-1464 |  | This study |
| pDIJ-1338F ^a^ | TAGTGAGTGTTTAGCTCAAGGA | 1338-1359 | 636 | This study |
| pDIJ-1973R ^a^ | GAATCACGCTGCGGTAT | 1973-1957 |  | This study |
| pDIJ-1815F ^a^ | TGATCAGTTATTGGGTCATGG | 1815-1835 | 601 | This study |
| pDIJ-2415R ^a^ | TCTTCGATGTATTCGTAATCTTCT | 2415-2392 |  | This study |
| pDIJ-2055F ^a^ | TCCGAGTCAATCAAGTGG | 2055-2072 | 606 | This study |
| pDIJ-2660R ^a^ | CATCGTTTGTGCAATGG | 2660-2644 |  | This study |
| pDIJ-2558F ^a^ | CCGAACGGATTGACCTT | 2558-2574 | 652 | This study |
| pDIJ-526R ^a^ | CGAACGCAGTGAGTTTG | 526-510 |  | This study |
| IS26-F | TAACAGCAAAGCTGCATAC | 393-411 | 205 | This study |
| IS26-R | TTTGCCATGATCGCATT | 597-581 |  | This study |
| orf513-209F | ATGGTTTCATGCGGGTT | 209-225 | 474 | [25] |
| orf513-683R | CTGAGGGTGTGAGCGAG | 689-667 |  | [24] |
| TN1-F | TCTGCTCCTTGAGAATGCAA | 57-76 | 189 | [23] |
| ISEcp1-246R | AAACTCGTTGACGAGGG | 246-230 |  | This study |
| Tn10s | TGCGAACTCGATATTTTACACG | 111-132 | 1135 | This study |
| Tn10as | GGTTGCAGCCACGAGTAAGT | 1265-1246 |  | This study |
| FII-1K for | CGCTGCTTATTTTCCCATTCC | 1046-1026 | 415 | G. Arlet ^b^ |
| FII-1Krev | CGTCCCGTTTTGATTTTTCCA | 632-652 |  | G. Arlet ^b^ |
| FII-2Kfor | GGGCTCATCACCTATCAGACT | 6153-6173 | 235 | G. Arlet ^b^ |
| FII-2Krev | GAAAACGCTCACGCACAAACC | 6387-6367 |  | G. Arlet ^b^ |
| repApLVPKfor | GTTGTGTCGTCTTTCCTCT | 218843-218825 | 606 | G. Arlet ^b^ |
| repApLVPKrev | GCTTCATCCAGTTGCTCTA | 218238-218256 |  | G. Arlet ^b^ |
| repAMET-1for | TCACCTACGGGCATCGCAA | 1339-1321 | 444 | G. Arlet ^b^ |
| repAMET-1rev | GAAATCCTCTGTTACCTCAAT | 896-916 |  | G. Arlet ^b^ |
| NewXXXfor | TTCAAGGTATGGGTCGGTATC | 23096-23076 | 348 | G. Arlet ^b^ |
| NewXXXrev | GTTTATCGGTTTATGTCGTAGC | 22749-22770 |  | G. Arlet ^b^ |

^a^ Positions indicated for the DNA mapping of *qnrD* plasmids are based on the nucleotide sequence of pDIJ09-518a (accession number HQ834472.1).

^b^ Personal data from Prof Guillaume Arlet.
