# Supplementary material for: Mobile Insertion Cassette Elements Found in Small Non-Transmissible Plasmids in Proteeae May Explain qnrD Mobilization
Source: PLoS One. 2014 Feb 4;9(2):e87801. doi: 10.1371/journal.pone.0087801 (PMC3913671; doi:10.1371/journal.pone.0087801)
Supplement: Table S2 — List of qnrD deposited in GenBank. (DOC) [file pone.0087801.s002.doc]

**Table S2.** List of *qnrD* –positive bacterial isolates or plasmids deposited in Genbank

| Bacterial strain | Plasmid reference | Plasmid size (bp) | Country | Authors | Genbank accession N° | Reference |
| --- | --- | --- | --- | --- | --- | --- |
| - | pHIRE-U1 | 2,682 | India | Kristiansson,E., *et al.* | HQ540672 | [29] |
| *Morganella morganii* CGH69 | pCGH69 | 2,683 | China | Zhang,S., *et al.* | JQ776510 | Unpublished |
| *Proteus mirabilis* CGH15 | pCGH15 | 2,683 | China | Zhang,S., *et al.* | JQ776508 | Unpublished |
| *Proteus mirabilis* CGS49 | pCGS49 | 2,683 | China | Zhang,S., *et al.* | JQ776507 | Unpublished |
| *Proteus mirabilis* CGP248 | pCGP248 | 2,683 | China | Zhang,S., *et al.* | JQ776503 | Unpublished |
| *Providencia rettgeri* GHS09-09 | pGHS09-09 | 2,683 | France | Guillard,T., *et al.* | HQ834473 | [18] |
| *Providencia rettgeri* DIJS09-518 | pDIJ09-518a | 2,683 | France | Guillard,T., *et al.* | HQ834472 | [18] |
| *Proteus mirabilis* T80 | pT80 | 2,687 | Italy | Mazzariol,A., *et al.* | JN18060 | [30] |
| *Morganella morganii* Vr831 | p831 | 2,684 | Italy | Mazzariol,A., *et al.* | JN18061 | [30] |
| *Proteus mirabilis*3M | P3M-2B | 5,903 | China | Chang M, *et al.* | JX514066 | Unpublished |
| *Escherichia coli* CGS13 | pCGS13 | 2,687 | China | Zhang,S., *et al.* | JQ776506 | Unpublished |
| *Escherichia coli* CGB40 | pCGB40 | 4,269 | China | Zhang,S., *et al.* | JQ776504 | Unpublished |
| *Escherichia coli* CGP169 | pCGP169 | 4,270 | China | Zhang,S., *et al.* | JQ776502 | Unpublished |
| *Escherichia coli* CGP246 | pCGP246 | 4,270 | China | Zhang,S., *et al.* | JQ776501 | Unpublished |
| *Citrobacter freundii* CGF41 | pCGF1 | 4,268 | China | Zhang,S., *et al.* | JQ776505 | Unpublished |
| *Klebsiella pneumoniae* CGH25 | pCGH25 | 4,270 | China | Zhang,S., *et al.* | JQ776509 | Unpublished |
| *Salmonella enterica* GSS-HN-2007057 | p2007057 | 4,270 | China | Cavaco,L.M., *et al.* | FJ228229 | [9] |
| *Proteus mirabilis* MPU 028P | - | - | Poland | Mokracka,J. *et al.* | JQ070958^a^ | Unpublished |
| *Proteus vulgaris* MPU 032P | - | - | Poland | Mokracka,J. *et al.* | JQ177060 ^a^ | Unpublished |
| *Morganella morganii* GZW7785 | - | - | China | Sun,J., *et al.* | JF776827 | Unpublished |
| *Proteus mirabilis* FSP49 | - | - | China | Sun,J., *et al.* | JF776822 | Unpublished |
| *Proteus mirabilis* GZP45 | - | - | China | Sun,J., *et al.* | JF776820 | Unpublished |
| *Proteus mirabilis* GZW1923 | - | - | China | Sun,J., *et al.* | JF776821 | Unpublished |
| *Escherichia coli* LHFL2 | - | - | China | Sun,J., *et al.* | JF776824 | Unpublished |
| *Escherichia coli* GZW2266 | - | - | China | Sun,J., *et al.* | JF776823 | Unpublished |
| *Citrobacter freundii* SG114 | - | - | China | Sun,J., *et al.* | JF776825 | Unpublished |
| *Citrobacter freundii* SPC10 | - | - | China | Sun,J., *et al.* | JF776826 | Unpublished |
| *Proteus vulgaris* QC48 | - | - | China | Zhao,J. *et al.*. | JN384197 | Unpublished |
| *Proteus vulgaris* QC46 | - | - | China | Zhao,J. *et al.* | JN384196 | Unpublished |
| *Escherichia coli* GDCA861 | pCA861 | ND | China | Yue,L., *et al.* | HM056768 | Unpublished |
| *Escherichia coli* GD026 | P026 | ND | China | Yue,L., *et al.* | HM056769 | Unpublished |
| *Escherichia coli* HN12 | - | - | China | Yun,Z.A., *et al.* | FJ606823 | Unpublished |
| *Escherichia coli* GP2009-036 | p1009036 | - | China | Zhu,H., *et al.* | GU233456 ^a^ | Unpublished |
| *Escherichia coli* G174 | - | - | China | Liu,J.-H., *et al.* | GU453932 | Unpublished |

^a^ partial *qnrD* sequence; -, no data
